# Advances in targeting the vacuolar proton-translocating ATPase (V-ATPase) for anti-fungal therapy

**DOI:** 10.3389/fphar.2014.00004

**Published:** 2014-01-27

**Authors:** Summer R. Hayek, Samuel A. Lee, Karlett J. Parra

**Affiliations:** ^1^Department of Biochemistry and Molecular Biology, School of Medicine, University of New Mexico Health Sciences CenterAlbuquerque, NM, USA; ^2^Department of Internal Medicine, School of Medicine, University of New Mexico Health Sciences CenterAlbuquerque, NM, USA; ^3^Section of Infectious Diseases, New Mexico Veterans Healthcare SystemAlbuquerque, NM, USA

**Keywords:** fungal V-ATPase, vacuolar proton pump, vacuolar acidification, pH homeostasis, *C. albicans* virulence, anti-fungal target

## Abstract

Vacuolar proton-translocating ATPase (V-ATPase) is a membrane-bound, multi-subunit enzyme that uses the energy of ATP hydrolysis to pump protons across membranes. V-ATPase activity is critical for pH homeostasis and organelle acidification as well as for generation of the membrane potential that drives secondary transporters and cellular metabolism. V-ATPase is highly conserved across species and is best characterized in the model fungus *Saccharomyces cerevisiae*. However, recent studies in mammals have identified significant alterations from fungi, particularly in the isoform composition of the 14 subunits and in the regulation of complex disassembly. These differences could be exploited for selectivity between fungi and humans and highlight the potential for V-ATPase as an anti-fungal drug target. *Candida albicans* is a major human fungal pathogen and causes fatality in 35% of systemic infections, even with anti-fungal treatment. The pathogenicity of *C. albicans* correlates with environmental, vacuolar, and cytoplasmic pH regulation, and V-ATPase appears to play a fundamental role in each of these processes. Genetic loss of V-ATPase in pathogenic fungi leads to defective virulence, and a comprehensive picture of the mechanisms involved is emerging. Recent studies have explored the practical utility of V-ATPase as an anti-fungal drug target in *C. albicans*, including pharmacological inhibition, azole therapy, and targeting of downstream pathways. This overview will discuss these studies as well as hypothetical ways to target V-ATPase and novel high-throughput methods for use in future drug discovery screens.

## V-ATPase PUMPS: STRUCTURE-FUNCTION AND MECHANISM OF CATALYSIS

### VACUOLAR H^**+**^-ATPase (V-ATPase) PUMPS ARE LARGE MULTI-SUBUNIT MOLECULAR MOTORS THAT COUPLE ACTIVE TRANSPORT OF PROTONS WITH ATP HYDROLYSIS TO ACIDIFY INTRACELLULAR COMPARTMENTS

V-ATPase pumps generate and sustain the distinctive organelle pH gradient of the endomembrane system that is necessary for Golgi, endosomal, vacuolar, and lysosomal functions (Kane, 2006; Forgac, 2007). Redistribution of protons from the cytosol to the lumen of acidic organelles by V-ATPase pumps is essential for organelle pH homeostasis. In fungi, V-ATPase also contributes to cytosolic pH regulation (Martinez-Munoz and Kane, 2008). Genetic and pharmacologic inactivation of V-ATPase pumps alters intracellular and extracellular pH. It disturbs numerous cellular processes including protein processing and sorting, protein secretion, receptor-mediated endocytosis, vesicular membrane trafficking, zymogen activation, and autophagy (Kane, 2006; Forgac, 2007).

**FIGURE 1:**
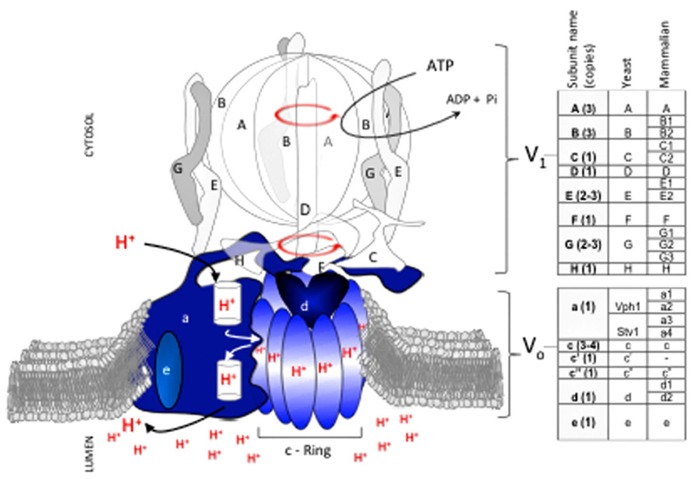
**V-ATPase subunit composition and mechanism of catalysis.** The V-ATPase proton pump acidifies the lumen of organelles in the endomembrane system of all eukaryotic cells. V-ATPase has 14 subunits that form two domains, V_1_ and V_o_. V_1_ (clear and gray subunits, A_3_B_3_CDE_3_FG_3_H) hydrolyzes ATP at the cytosolic side of the membrane, and V_o_ (blue subunits, ac_3__-__4_c’c”de) translocates protons. Transport of protons against a concentration gradient entails a rotational mechanism. Hydrolysis of 3 ATP in the V_1_A catalytic subunits drives rotation of a shaft (subunits D, F) that penetrates V_1_ and is bound to a rotating proteolipid ring structure in V_o_ formed by subunits c_(3__-__4)_c’c” (c-ring) that together with subunit V_o_a forms the path for proton transport. With the exception of subunit V_o_a, which exists in two isoforms (Vph1p and Stv1p), each V-ATPase subunit is encoded by a single gene in fungi. In contrast, multiple isoforms (two to four) exist for most subunits of the mammalian V-ATPase. Subunit V_o_c′ is absent in mammals.

The vast majority of structural and mechanistic data on eukaryotic V-ATPases available have been collected in *Saccharomyces* *cerevisiae*. V-ATPase proton transport requires structural and functional coupling of a peripheral domain (V_1_) with a membrane-embedded domain (V_o_; **Figure 1**). Coupling involves an intricate mechanism that uses relative rotation of subunits in *V*_1_ and *V*_o_ (Sun-Wada et al., 2003; Forgac, 2007). During catalysis, hydrolysis of ATP within the protuberant structure of *V*_1_ drives rotation of a central stalk (the rotor’s shaft) located near the catalytic sites. The rotating central stalk is connected to a hydrophobic ring of proteolipid-like subunits in *V*_o_ (c-ring). During rotation, each subunit of the c-ring has one essential glutamate residue that accepts a proton from the cytosol and transfers it to the organelle’s lumen against a concentration gradient.

Important changes occurred as V-ATPase evolved from *S. cerevisiae* to humans. For example, the c-ring of all fungal species contains a combination of three subunits (V_o_c, V_o_c′, V_o_c^′′^) whereas mammalian c-rings lack the V_o_c′ subunit. Phylogenic analysis by Finnigan et al. (2012) suggests that the fungal V_o_c and V_o_c′ subunits evolved from a gene duplication in an ancestral gene that was common to all fungal species. Intriguingly, the functions of V_o_c and V_o_c′ in fungi, particularly the binding capabilities of each subunit, appear to have degenerated from the common ancestor (Finnigan et al., 2012). This example of constructive neutral evolution suggests that the complexity of the V-ATPase machine may have been driven in part by loss-of-function processes(Doolittle, 2012).

In general, the complexity of V-ATPase increased as species evolved further from fungi. Mammals developed multiple tissue and membrane specific isoforms for most V_1_ and V_o_ subunits (**Figure 1**; Marshansky and Futai, 2008; Sun-Wada and Wada, 2010; Toei et al., 2010). In contrast, only one subunit (V_o_a) of V-ATPase in budding yeast species such as *S. cerevisiae* has functional homologues (Manolson et al., 1994), and non-budding fungi such as *Neurospora crassa* contain only single subunit isoforms (Chavez et al., 2006).

The sequence conservation between human and *S. cerevisiae* subunits is 31–41% identity and 51–60% similarity, depending on the subunit and isoform (Rahman et al., 2013). This relatively low sequence conservation may explain differences in binding affinity between V-ATPase subunits from human (Rahman et al., 2013) and *S. cerevisiae* (Oot and Wilkens, 2012); it may also fine tune V-ATPase activity and determine regulatory mechanisms in response to diverse cellular signals and environments. Although information describing the topological arrangements of the human V_1_V_o_ complex is not available, the V-ATPase overall structure and its sophisticated mechanism of rotational catalysis are likely conserved from *S. cerevisiae*.

This review focuses on fungal V-ATPases with an emphasis on the human fungal pathogen *Candida albicans*. We summarize our current understanding of the roles of V-ATPase in pathogenicity as well as its antifungal drug targeting potential. Because V-ATPase subunit structure and composition, assembly and regulation, and multiple downstream cellular functions are best studied in the fungus *S. cerevisiae* (Kane, 2006, 2007), we also refer to *S.* *cerevisiae* and other fungi throughout this review.

## *Candida albicans* IS THE PRIMARY HUMAN FUNGAL PATHOGEN

*Candida albicans* is the most frequently diagnosed fungal pathogen and is the fourth leading cause of hospital-acquired bloodstream infections in North America (Klotz et al., 2007). *C. albicans* is normally a harmless commensal in the oral cavity, digestive tract, and genital region of healthy people but is also associated with superficial infections. *C. albicans* can enter the bloodstream following tissue damage or the formation of fungal biofilms on medical implants, leading to sepsis, and organ failure. These severe cases of systemic candidiasis are most common in patients undergoing immunosuppressive therapy or that are otherwise immunocompromised (Pfaller and Diekema, 2007). Critically, patient mortality rates can reach 35% even with anti-fungal treatment (Horn et al., 2009).

Invasive infection due to *C. albicans* is a multifactorial process that relies on numerous virulence factors to control pathogenesis. *C. albicans* can exist as either a unicellular yeast or a filamentous hyphae. This morphological dimorphism contributes to virulence, as the yeast form is considered nonpathogenic while the hyphal form induces damage and invasion of host tissue (Sudbery, 2011). *C. albicans* also secretes serine aspartyl proteinases and lipases that are involved in nutrient acquisition, host cell degradation, and immune evasion (Naglik et al., 2003). Other *C. albicans* virulence pathways include iron acquisition from hemoglobin, protection against reactive oxygen species, expression of adhesion molecules, and formation of biofilms. Together, these pathways facilitate host cell invasion and protect against the host immune response (Karkowska-Kuleta et al., 2009).

## *Candida albicans* PATHOGENICITY CORRELATES WITH pH REGULATION, SUGGESTING THAT V-ATPase MAY PLAY A FUNDAMENTAL ROLE IN FUNGAL VIRULENCE

### *Candida albicans* VIRULENCE IS REGULATED BY pH AT THE EXTRACELLULAR, VACUOLAR, AND CYTOPLASMIC LEVEL

Extracellular pH controls the morphological dimorphism of *C. albicans*. The non-pathogenic yeast form of the fungus grows preferentially under acidic environmental conditions while increasing extracellular pH triggers hyphal growth and increased virulence (Sudbery, 2011). *C. albicans* has developed mechanisms that allow it to rapidly respond to environmental pH changes, and mutants lacking the ability to sense extracellular pH display reduced virulence (Davis, 2009).

Vacuolar pH is important for numerous aspects of *C. albicans* physiology and virulence. Preservation of a proton gradient across the vacuolar membrane is critical for general cellular metabolism, including receptor-mediated endocytosis, intracellular membrane trafficking, pro-hormone processing, protein degradation, uptake of small molecules, and storage and detoxification of metabolites and ions (Veses et al., 2008). Additionally, both activation and secretion of the proteinase and lipase virulence factors and activity of the enzymes themselves require optimal vacuolar pH (Naglik et al., 2003). Numerous *C. albicans* mutants that display abnormal vacuolar alkalinization also display reduced filamentation and defective *in vivo* virulence (Bruckmann et al., 2000; Jia et al., 2002; Eck et al., 2005; Poltermann et al., 2005; Palanisamy et al., 2010; Zhang et al., 2010; Patenaude et al., 2013; Rane et al., 2013). The azole class of anti-fungal drugs also functions in part through disruption of vacuolar acidification (Zhang et al., 2010).

Finally, cytoplasmic pH contributes to filament formation during *C. albicans* virulence. Germ tube formation, the precursor step to hyphal formation, requires alkalinization of the cytoplasm (Stewart et al., 1988). In fungi, cytosolic pH is regulated via the Pma1p plasma membrane proton transporter, which pumps protons out of the cell and into the extracellular space to maintain a neutral-to-alkaline cytosol and an acidic extracellular environment (Monk et al., 1991). The importance of Pma1p activity and cytosolic alkalinization in *C. albicans* virulence is illustrated by studies showing that Pma1p activity and expression is upregulated during filamentation (Kaur and Mishra, 1991; Monk et al., 1993). Furthermore, *C. albicans* mutants that cannot properly alkalinize their cytosol in response to filamentation cues are avirulent (Stewart et al., 1988, 1989; Mahanty et al., 1990).

The central importance of pH regulation in *C. albicans* virulence makes V-ATPase an attractive target for anti-fungal therapy (Parra, 2012). In addition to its critical role in vacuolar pH homeostasis (Kane, 2006; Tarsio et al., 2011), V-ATPase is a known regulator of Pma1p activity in *S. cerevisiae*. V-ATPase mutants display abnormally acidified cytosol due to a lack of properly localized or functional Pma1p (Perzov et al., 2000; Martinez-Munoz and Kane, 2008; Huang and Chang, 2011). Together, these data suggest that V-ATPase may contribute to numerous virulence pathways in *C. albicans*, including vacuolar function and during the germ tube-to-hyphae morphological transition*.* Next, we summarize studies that have examined the role of V-ATPase in fungal pathogenesis using genetic loss-of-function studies.

## GENETIC LOSS OF V-ATPase IN PATHOGENIC FUNGI AFFECTS VIRULENCE

### GENETIC STUDIES IN VARIOUS PATHOGENIC FUNGI HAVE ESTABLISHED A LINK BETWEEN V-ATPase ACTIVITY, VACUOLAR ACIDIFICATION, AND FUNGAL VIRULENCE

Hilty et al. (2008) removed *VMA1* (the V_1_A subunit of V-ATPase) from the *Histoplasma capsulatum* genome and demonstrated that V_1_A is required for iron sequestration, replication in macrophages, and growth as a mold. The *vma1* mutants were also avirulent in a mouse model of histoplasmosis (Hilty et al., 2008). In *Cryptococcus neoformans,* loss of the V_o_a subunit via deletion of *VPH1* leads to defective production of capsule, laccase, and urease, three virulence factors required for *C. neoformans* infectivity. These *vph1* mutants also displayed defective *in vivo* virulence in a murine model of meningo-encephalitis (Erickson et al., 2001). These findings lend credence to the idea that V-ATPase plays a critical role in the maintenance of fungal virulence.

In contrast, studies in *Aspergillus fumigatus* suggest that host V-ATPase plays an important protective role during immune defense against fungal pathogens. *A. fumigatus* is the primary causative agent of life-threatening invasive bronchopulmonary aspergillosis. While host V-ATPase is critical for phagolysosomal acidification and pathogen killing under non-pathogenic conditions, infective variants of *A. fumigatus* prevent acidification of phagolysosomes and allow for *A. fumigatus* germination and immune escape (Ibrahim-Granet et al., 2003). Notably, work by Slesiona et al. demonstrated that less virulent *Aspergillus* variants can be made virulent via pharmacological inhibition of host V-ATPase resulting in loss of phagolysosomal acidification (Slesiona et al., 2012). These findings suggest that fungal pathogens may inactivate host V-ATPase pathways during immune evasion and suggest that the balance of pathogen versus host V-ATPase activity is critical for determining virulence.

Studies specifically examining virulence in *C. albicans* have further solidified the importance of V-ATPase in this process. In the iron-deplete conditions of host tissue, *C. albicans* must extract iron from hemoglobin for survival. Weissman et al. (2008) demonstrated that null mutants of *vma11* (subunit V_o_c′) are deficient in iron acquisition. Upon loss of *VMA7* (subunit V_1_F) in *C. albicans*, Poltermann et al. (2005) noted defects in *in vitro* filamentation and *in vivo* virulence during systemic candidiasis. The authors further connected these defects to biochemical phenotypes including vacuolar alkalinization, pH-dependent growth, and sensitivity to metal ions (Poltermann et al., 2005). Recently, our laboratories demonstrated that inducible loss of *VMA3* (subunit V_o_c of the c-ring) in *C. albicans* results in alkaline vacuoles with fission defects, leading to reduced protease and lipase secretion, defective filamentation, and ineffective macrophage killing (Rane et al., 2013). Unpublished studies from our laboratories have yielded similar phenotypes following the inducible loss of *VMA2* (subunit V_1_B).

**FIGURE 2:**
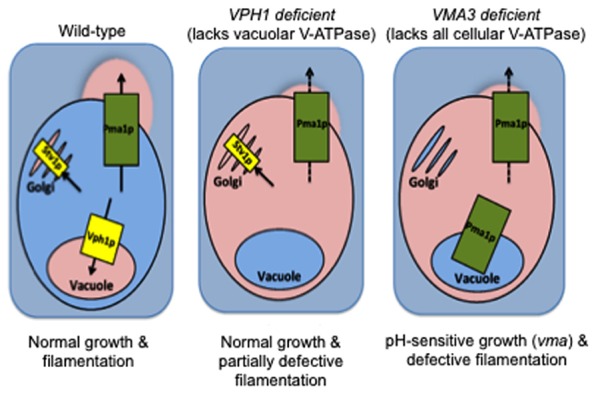
**V-ATPase in non-vacuolar organelles (Stv1p-containing complexes) plays a yet unknown role in *C. albicans* virulence**. Wild-type: When functional, V-ATPase-mediated acidification of vacuoles, Golgi, and secretory vesicles maintains organelle pH and supports traffic of Pma1p to the cell surface for proton efflux and maintenance of an alkaline cytosol. *VPH1 deficient*: *C. albicans* grows normally at neutral pH when only Vph1p-containing V-ATPase complexes (vacuolar membrane) are missing. Only modest filamentation defects are obvious, despite the concomitant vacuolar alkalinization and defective Pma1p activity (Raines et al., 2013 and unpublished results). *VMA3 deficient*: Vacuolar alkalinization and defective Pma1p activity occur at levels equal to that of *VPH1* deficient cells when all V-ATPase function is missing (Stv1p- and Vph1-containing V-ATPase complexes; Rane et al., 2013 and unpublished results). However, *VMA3* deficient *C. albicans* exhibits growth defects at neutral pH and severely reduced filamentation under these conditions. We therefore hypothesize that the presence of Stv1p-containing V-ATPase in non-vacuolar organelles maintains virulence in the face of defective vacuolar and cytoplasmic pH homeostasis. Pink = acidic/acidified, blue = alkaline/alkalinized.

In *C. albicans*, the V_o_a subunit is the only subunit within the enzyme complex that is encoded by multiple isoforms: *VPH1* localizes V-ATPase to vacuoles while *STV1* traffics V-ATPase to the Golgi and endocytic organelles (Patenaude et al., 2013; Raines et al., 2013). This difference was recently exploited to study the contribution of organelle-specific V-ATPase to *C. albicans* virulence. Both our laboratories and Patenaude et al. (2013) showed that loss of *VPH1* but not *STV1* leads to vacuolar alkalinization, abnormal vacuolar morphology, and defective metal ion sequestration (Raines et al., 2013). Our study noted that although these biochemical defects contributed to reduced protease and lipase secretion, the *vph1* mutants displayed only modest filamentation defects and wild-type levels of biofilm formation and macrophage killing (Raines et al., 2013). Our results suggest that vacuolar acidification is dispensable during certain *C. albicans* virulence pathways. Furthermore, Stv1p-containing V-ATPase complexes may play novel roles in pathogenesis (**Figure 2**). In contrast, the *vph1* mutant cells used in the Patenaude et al. (2013) study displayed defective filamentation, reduced damage to epithelial and macrophage host cells, and avirulence during systemic *in vivo* infection. These results suggest that vacuolar acidification is essential for all forms of *C. albicans* virulence. The explanation for these disparate results remains to be determined but likely centers around differences in strain background or methodology. Nonetheless, these studies raise the fascinating possibility that organelle-specific V-ATPase activity can be modulated to control specific *C. albicans* virulence pathways.

## CURRENT UTILITY OF V-ATPase AS AN ANTI-FUNGAL DRUG TARGET

The recent emergence of multidrug resistant strains of *C. albicans* has made the development of novel classes of anti-fungal drugs paramount (Hameed and Fatima, 2013). V-ATPase is an attractive target for drug discovery, given the numerous lines of genetic evidence supporting a critical role for V-ATPase in *C. albicans* virulence. We next consider the feasibility of V-ATPase as an anti-fungal drug target via an overview of three currently available methods that utilize V-ATPase-related mechanisms: pharmacological inhibition of V-ATPase, azole therapy, and Pma1p inhibition.

### PHARMACOLOGICAL INHIBITION OF V-ATPase

V-ATPase inhibitors have been used for over 20 years to study the function and mechanism of V-ATPase activity in organisms ranging from fungi to humans. As of 2009, eight types of V-ATPase inhibitors had been described, including the best characterized plecomacrolide class (Huss and Wieczorek, 2009). The plecomacrolides include bafilomycin and concanamycin, which are antibiotics that bind to the V_o_c subunit of V-ATPase to prevent c-ring rotation and interfere with ATP hydrolysis and proton transport simultaneously (Bowman et al., 1988; Drose et al., 1993). Notably, both bafilomycin and concanamycin have been shown to inhibit V-ATPase from *C. albicans* (Calvert and Sanders, 1995; Chan et al., 2012). However, none of the molecules belonging to the aforementioned eight classes of V-ATPase inhibitors are currently used to treat *C. albicans* infection in a clinical setting; many cannot differentiate between the fungal target and mammalian host V-ATPase (Bowman and Bowman, 2005; Huss and Wieczorek, 2009).

Recently, our laboratory developed a high-throughput screening method to identify new V-ATPase inhibitors in *S. cerevisiae* (Chan et al., 2012). In this method, *S. cerevisiae* cells are transformed with the gene for pHluorin, a pH-sensitive version of GFP (Brett et al., 2005). The fluorescence intensity of pHluorin can be used to identify molecules that acidify the cytosol of *S. cerevisiae* following V-ATPase inhibition. We screened the Prestwick Chemical Library (a collection of 1120 off-patent drug compounds) using this method and identified alexidine dihydrochloride and thonzonium bromide as novel and specific V-ATPase inhibitors (Chan et al., 2012). Cells treated with these drugs showed a V-ATPase-specific pH-sensitive growth phenotype (*vma* phenotype) exclusive of fungi species. These inhibitors belong to a unique class of V-ATPase uncouplers that inhibit proton transport without affecting ATP hydrolysis. We also confirmed that disulfiram, a compound identified previously by a high-throughput screening method that identifies drugs that alkalinize vacuoles (Johnson et al., 2010), inhibits V-ATPase activity via the pHluorin screening method.

Alexidine dihydrochloride, thonzonium bromide, and disulfiram also inhibit V-ATPase in isolated *C. albicans* vacuoles. Alexidine dihydrochloride and thonzonium bromide also cause general cellular toxicity in intact *C. albicans* cells (Chan et al., 2012). These data suggest that these pre-existing drugs could be repurposed as anti-fungal therapies. Indeed, the majority of compounds in the Prestwick Chemical Library have known safety and toxicity profiles (Wermuth, 2004), making drug repurposing a less daunting task than *de novo* drug discovery.

### AZOLE THERAPY

Azoles are a class of antifungal agents that are commonly used to treat fungal infections in the clinical setting. Azoles inhibit C-14α-demethylase, an enzyme necessary for the conversion of lanosterol to ergosterol, an essential component of fungal membranes (Chapman et al., 2008). At least four ergosterol biosynthesis genes (*ERG3*, *ERG4*, *ERG5*, and *ERG11*) are affected by azoles. The general membrane disruption resulting from azole treatment alters the activity of several membrane-bound transporters and channels including those associated with the transport of amino acids and other nutrients, chitin synthesis, and mitochondrial oxidation (Vanden Bossche, 1985; Barrett-Bee et al., 1991; Georgopapadakou and Walsh, 1996).

For some time, it was assumed that azoles caused fungal toxicity primarily through damage to the cell membrane resulting in cell permeability and lysis (Chapman et al., 2008). However, recent work has uncovered a critical role for ergosterol in V-ATPase function. In *S. cerevisiae*, mutants defective in ergosterol biosynthesis display alkaline vacuoles and the *vma* phenotype (Zhang et al., 2010). Surprisingly, the V-ATPase holoenzyme is properly assembled at the vacuolar membrane despite ergosterol depletion. The mutant phenotypes instead appear to result from reduced ATP hydrolysis and proton pumping within the V-ATPase. Fluconazole treatment of *C. albicans* cells also results in alkaline vacuoles (Zhang et al., 2010), further solidifying that azoles function in part through inhibition of V-ATPase.

These studies by Zhang et al. (2010) have identified a new regulatory pathway involved in V-ATPase function as well as elucidated a novel mechanism underlying azole toxicity in *C. albicans*. From a therapeutic standpoint, these studies provide proof-of-principle that anti-V-ATPase agents could work in the clinical setting. Importantly, drugs targeting V-ATPase may act mechanistically similar to azoles while bypassing the multidrug resistance that has plagued current anti-fungal therapies.

### Pma1p INHIBITION

Given the interplay between V-ATPase and Pma1p in *S. cerevisiae* (Perzov et al., 2000; Martinez-Munoz and Kane, 2008; Huang and Chang, 2011) and *C. albicans* (unpublished results), anti-fungal therapies that rely on Pma1p inhibition may affect pathways downstream of V-ATPase function. Indeed, Bowman et al. (1997) demonstrated that 64 of 66 mutations that rescued concanamycin A-mediated V-ATPase inhibition in *N. crassa* were localized to the *PMA1* gene. The synthetic organoselenium compound ebselen is a known inhibitor of Pma1p (Chan et al., 2007; Billack et al., 2009). Ebselen exhibits anti-fungal activity against wild-type strains of *C. albicans* (Soteropoulos et al., 2000; Bouhafs and Jarstrand, 2002; Wojtowicz et al., 2004) as well as fluconazole-resistant strains (Billack et al., 2009). These results again demonstrate the precedence that targeting components of the V-ATPase pathway, even via a downstream element such as Pma1p, can circumvent the drug resistance that has developed against presently available therapies.

## HYPOTHETICAL APPROACHES FOR TARGETING V-ATPase

### THE DEVELOPMENT OF NEW ANTI-FUNGAL TREATMENTS AGAINST V-ATPase MUST INVOLVE NOVEL STRATEGIES FOR IDENTIFYING THERAPEUTIC TARGETS

Selectivity for fungal pathogens over innocuous host tissue is one critical targeting factor to consider. The V_o_c′ subunit of V-ATPase, encoded by *VMA11*, is a promising candidate target (Parra, 2012). The V_o_c′ subunit is found specifically in fungi and lacks a mammalian homolog (**Figure 1**). Promisingly, previous studies have demonstrated that *C. albicans* null mutants of *vma11* do not properly acquire iron from hemoglobin, a critical virulence factor within host tissue (Weissman et al., 2008). Future structural studies of the V_o_c′ subunit will aid in the rational design of a fungal-specific anti-V-ATPase drug.

Most fungal V-ATPase subunits are encoded by a single gene; the V_o_a subunit is the only subunit encoded by two isoforms. Mammalian V-ATPases are strikingly different, with seven different subunits displaying isoform variation (Jefferies et al., 2008; **Figure 1**). This drastic difference between fungi and mammals could be exploited for fungal-specific drug development. For example, the V_1_C subunit exists as three isoforms in mammals. If an inhibitor is designed to specifically target a domain of fungal V_1_C that is missing from one or more of the mammalian isoforms, all cellular V-ATPase activity will cease in the fungal pathogen while the non-targeted mammalian isoforms compensate and maintain V-ATPase activity in the host cell. This strategy will require high-resolution structures of all fungal and mammalian isoforms and is therefore a longer-range possibility. However, it remains a fascinating option for fungal-specific drug targeting.

V-ATPase activity is regulated in part by reversible disassembly, the process by which the V_1_ and V_o_ sectors separate from one another to prevent ATP hydrolysis and proton transport (Kane and Parra, 2000; Kane, 2006). Reversible disassembly is triggered primarily by glucose deprivation, although extracellular pH and salt stress are thought to contribute to the process. In mammals, cell-specific regulatory pathways also control disassembly in a tissue-specific manner (Kane, 2012). Disassembly in *C. albicans* has not been studied, but conserved similarities between *S. cerevisiae* and mammals suggest *C. albicans* will utilize similar pathways. Notably, the V_o_a subunit and organelle environment appear to regulate disassembly in *S. cerevisiae*; Vph1p-containing complexes disassociate upon glucose deprivation while Stv1p-containing complexes are less sensitive (Kawasaki-Nishi et al., 2001). In contrast, mammalian cells contain four V_o_a isoforms localized to different tissues. This suggests that differences in isoform structure and interaction, localization, or susceptibility to environmental cues could be exploited for fungal-specific drug development as described previously. Alternatively, indirectly targeting the regulator proteins involved in disassembly may allow for fungal-specific inhibition of V-ATPase activity (Kane, 2012).

Finally, Pma1p is a fungal-specific protein with no known mammalian homologs. Simultaneous targeting of both V-ATPase and Pma1p should therefore disrupt both vacuolar and cytoplasmic pH homeostasis in fungi versus vacuolar pH alone in mammalian cells. This dual approach may thereby prove more detrimental to the fungal pathogen than the host cell. This method is also advantageous in that it can be achieved either by creation of a novel V-ATPase/Pma1p dual inhibitor or by administration of an anti-fungal drug cocktail consisting of presently available therapies. Additionally, as Pma1p is a plasma membrane protein with access to the extracellular space, anti-Pma1p drugs may function regardless of accumulation within the fungal cell. This possibility is highly advantageous, as drug uptake often limits efficacy in fungi due to a lack of specific uptake systems.

## A NOVEL HIGH-THROUGHPUT METHOD FOR FUTURE V-ATPase DRUG DISCOVERY IN *C. albicans*

Our need for anti-V-ATPase therapies grows increasingly critical as we improve our understanding of the function of V-ATPase in fungal virulence. However, direct screening for anti-V-ATPase drugs in *C. albicans* is typically hindered by the non-availability of high-throughput screening tools in the pathogenic fungi. We previously demonstrated the practical utility of measuring cytosolic pH as a surrogate for V-ATPase function in a high-throughput screen for inhibitors of *S. cerevisiae* V-ATPase (Chan et al., 2012). This method uses pHluorin, a pH-sensitive GFP construct that can be stably transformed, thereby avoiding expensive and time-consuming single-use dyes. Recently, Ullah et al. (2013) were the first to successfully express pHluorin in a pathogenic fungus, *C. glabrata*. However, non-canonical codon use precludes the direct application of the existing pHluorin construct in *C. albicans*.

Our lab is currently working to create a pHluorin construct optimized for *C. albicans* (CapHluorin) that will enable easy, inexpensive, and high-throughput measurement of cytosolic pH for use in screening for V-ATPase inhibitors. Additionally, alkalinization of the cytoplasm is a general determinant of fungal virulence (Stewart et al., 1988), and CapHluorin-mediated drug screens may result in the rapid discovery of new anti-fungal therapies that function independently of V-ATPase. Previous studies in *S. cerevisiae* proved the feasibility of modifying pHluorin for targeting to specific cellular compartments such as the Golgi (Tarsio et al., 2011). CapHluorin will facilitate similar studies to examine the importance of organelle-specific V-ATPase function and pH homeostasis in *C. albicans*. Importantly, the CapHluorin construct optimized for non-canonical codon use will enable cytosolic pH measurements not just in *C. albicans*, but in all fungal species that utilize the CTG clade, including the human pathogens *C. dubliniensis*, *C. tropicalis*, *C. parapsilosis*, and *C. lusitaniae* (Papon et al., 2012).

## KEY CONCEPTS

### VACUOLAR H^**+**^-ATPase (V-ATPase) PUMPS ARE LARGE MULTI-SUBUNIT MOLECULAR MOTORS THAT COUPLE ACTIVE TRANSPORT OF PROTONS WITH ATP HYDROLYSIS

V-ATPase activity is required for acidification of intracellular compartments and generates and sustains the pH gradient required for function of the endomembrane system organelles.

### *Candida albicans* IS THE MOST FREQUENTLY DIAGNOSED FUNGAL PATHOGEN

Although it exists commensally with humans, it can cause life-threatening blood infections under optimal pathogenic conditions. *C. albicans* has developed resistance to many currently available anti-fungal drugs, and mortality rates can reach 35% even with proper treatment. There is therefore a dire need to develop new anti-fungal therapies.

### *Candida albicans* VIRULENCE IS REGULATED BY pH AT THE EXTRACELLULAR, VACUOLAR, AND CYTOPLASMIC LEVEL

Extracellular pH triggers a morphological switch to the pathogenic form of the fungus. Vacuolar acidification allows for activation/secretion of virulence enzymes. Cytoplasmic alkalinization precedes the formation of germ tubes during *C. albicans* filamentation.

### GENETIC STUDIES IN VARIOUS PATHOGENIC FUNGI HAVE ESTABLISHED A LINK BETWEEN V-ATPase ACTIVITY, VACUOLAR ACIDIFICATION, AND FUNGAL VIRULENCE

Studies in *Histoplasma capsulatum*, *Cryptococcus neoformans*, and *Candida albicans* have demonstrated that loss of all cellular V-ATPase leads to alkaline vacuoles and a host of *in vitro* and *in vivo* virulence defects.

### V-ATPase IS AN ATTRACTIVE TARGET FOR DRUG DISCOVERY

Current anti-fungal therapies that involve the V-ATPase pathway include direct pharmacological inhibition of V-ATPase, the clinically prescribed azole class of drugs, and Pma1p inhibitors. However, the emergence of multidrug resistant strains of *C. albicans* makes additional drug discovery imperative.

### THE DEVELOPMENT OF NEW ANTI-FUNGAL TREATMENTS AGAINST V-ATPase MUST INVOLVE NOVEL STRATEGIES FOR IDENTIFYING THERAPEUTIC TARGETS

Such strategies include targeting the fungal-specific V_o_c′ subunit, utilizing differences in isoform composition and complex disassembly between fungi and mammals, dual V-ATPase/Pma1p inhibition, and creation of a high-throughput screen in *C. albicans*.

## Conflict of Interest Statement

The authors declare that the research was conducted in the absence of any commercial or financial relationships that could be construed as a potential conflict of interest.
